# Misinformation, Trust, and Use of Ivermectin and Hydroxychloroquine for COVID-19

**DOI:** 10.1001/jamahealthforum.2023.3257

**Published:** 2023-09-29

**Authors:** Roy H. Perlis, Kristin Lunz Trujillo, Jon Green, Alauna Safarpour, James N. Druckman, Mauricio Santillana, Katherine Ognyanova, David Lazer

**Affiliations:** 1 Department of Psychiatry, Massachusetts General Hospital, Boston; 2 Department of Psychiatry, Harvard Medical School, Boston, Massachusetts; 3 Associate Editor, *JAMA Network Open*; 4 Department of Political Science, Northeastern University, Boston, Massachusetts; 5 John F. Kennedy School of Government and Department of Government, Harvard University, Cambridge, Massachusetts; 6 Department of Political Science, Northwestern University, Evanston, Illinois; 7 Department of Communication, School of Communication and Information, Rutgers University, New Brunswick, New Jersey

## Abstract

**Importance:**

The COVID-19 pandemic has been notable for the widespread dissemination of misinformation regarding the virus and appropriate treatment.

**Objective:**

To quantify the prevalence of non–evidence-based treatment for COVID-19 in the US and the association between such treatment and endorsement of misinformation as well as lack of trust in physicians and scientists.

**Design, Setting, and Participants:**

This single-wave, population-based, nonprobability internet survey study was conducted between December 22, 2022, and January 16, 2023, in US residents 18 years or older who reported prior COVID-19 infection.

**Main Outcome and Measure:**

Self-reported use of ivermectin or hydroxychloroquine, endorsing false statements related to COVID-19 vaccination, self-reported trust in various institutions, conspiratorial thinking measured by the American Conspiracy Thinking Scale, and news sources.

**Results:**

A total of 13 438 individuals (mean [SD] age, 42.7 [16.1] years; 9150 [68.1%] female and 4288 [31.9%] male) who reported prior COVID-19 infection were included in this study. In this cohort, 799 (5.9%) reported prior use of hydroxychloroquine (527 [3.9%]) or ivermectin (440 [3.3%]). In regression models including sociodemographic features as well as political affiliation, those who endorsed at least 1 item of COVID-19 vaccine misinformation were more likely to receive non–evidence-based medication (adjusted odds ratio [OR], 2.86; 95% CI, 2.28-3.58). Those reporting trust in physicians and hospitals (adjusted OR, 0.74; 95% CI, 0.56-0.98) and in scientists (adjusted OR, 0.63; 95% CI, 0.51-0.79) were less likely to receive non–evidence-based medication. Respondents reporting trust in social media (adjusted OR, 2.39; 95% CI, 2.00-2.87) and in Donald Trump (adjusted OR, 2.97; 95% CI, 2.34-3.78) were more likely to have taken non–evidence-based medication. Individuals with greater scores on the American Conspiracy Thinking Scale were more likely to have received non–evidence-based medications (unadjusted OR, 1.09; 95% CI, 1.06-1.11; adjusted OR, 1.10; 95% CI, 1.07-1.13).

**Conclusions and Relevance:**

In this survey study of US adults, endorsement of misinformation about the COVID-19 pandemic, lack of trust in physicians or scientists, conspiracy-mindedness, and the nature of news sources were associated with receiving non–evidence-based treatment for COVID-19. These results suggest that the potential harms of misinformation may extend to the use of ineffective and potentially toxic treatments in addition to avoidance of health-promoting behaviors.

## Key Points

**Question:**

How much were beliefs in misinformation and trust in institutions associated with individuals’ use of non–evidence-based treatments for COVID-19, including ivermectin and hydroxychloroquine?

**Findings:**

Among 13 438 adults in a 50-state US survey study who reported probable or definite COVID-19 infection, 6% reported use of either ivermectin or hydroxychloroquine. Those who endorsed COVID-19 vaccine–related misinformation, had lower trust in hospitals and physicians and lower trust in scientists, and exhibited greater belief in conspiracy theories were more likely to report using one of these medicines.

**Meaning:**

Approximately 1 in 20 people with probable COVID-19 reported using a non–evidence-based treatment, and these individuals were more likely to exhibit specific deleterious beliefs and attitudes not captured by political affiliation.

## Teaser

This survey study examines the association between non–evidence-based treatment for COVID-19 and endorsement of misinformation and trust in institutions among US residents with prior COVID-19 infection.

## Introduction

The antiparasitic agents ivermectin and hydroxychloroquine were both touted as cures for the SARS-CoV-2 virus, in the absence of evidence of efficacy. Despite multiple US Food and Drug Administration (FDA) statements clarifying that these medicines are not indicated for COVID-19,^1^ prescribing increased substantially in the US, Canada, and Australia in the first year of the pandemic^2,3,4^; 1 study^3^ showed an association between prescribing and county-level support for Donald Trump. Beyond a lack of efficacy, such prescriptions were associated with risk of toxic effects.^5^ Their use thus introduced inefficiencies and expense as well as potential adverse effects on health and may help explain the observed association between more misinformation-prone counties and greater COVID-19 death rates.^6^

In light of the potential consequences of non–evidence-based treatment, to inform future pandemic responses, we sought to better understand the prevalence of such treatment during the COVID-19 pandemic and the features associated with its use. Prior studies^1,2,3,4^ using prescription databases may not capture overall prevalence of non–evidence-based treatment use if these medications were acquired in other ways and have not described the full range of sociodemographic features associated with their use.

Beyond such features, we sought to understand other characteristics of individuals that might be associated with risk of non–evidence-based treatment use. In particular, the rapid dissemination of demonstrably false information about COVID-19 has been a notable feature of the pandemic.^7^ From the source of the pandemic,^8^ to its means of spread, to its potential impact, false or misleading statements have been widespread on social media^9^ as well as some traditional news sources. Among the most prominent types of misinformation have been statements concerning the off-label treatment of COVID-19.

We therefore also investigated the association between endorsing misinformation about the COVID-19 vaccine and pursuing these treatments, hypothesizing that susceptibility to misinformation would also be reflected in treatment choice. More broadly, we explored trust in institutions, reasoning that those with lesser trust in health care institutions might be more likely to pursue non–evidence-based treatments. We examined the extent to which conspiratorial thinking in general is associated with choosing these treatments. Finally, we investigated whether source of news about COVID-19 was associated with use of non–evidence-based medication. In all of these analyses, we modeled the effects of political affiliation to understand whether any observed associations could be explained by such affiliation alone.

## Methods

### Study Design

For this survey study, we used data from wave 26 of the COVID States Project, an academic consortium that has conducted a 50-state internet survey approximately every 4 to 8 weeks since spring 2020. The survey wave used in this study was conducted between December 22, 2022, and January 16, 2023. The survey uses a nonprobability sampling^10^ design, applying state-level representative quotas for race and ethnicity, age, and gender. Survey respondents are 18 years or older, reside in the US, and are asked to provide written informed consent before beginning the survey. The study was classified as exempt by the institutional review board of Harvard University because it represents minimal risk to participants. We describe all results according to the American Association for Public Opinion Research (AAPOR) reporting guideline.^11^

### Measures

Participants were asked whether they had previously been diagnosed with COVID-19 or believed they had previously been infected. The primary analytic cohort included all individuals who answered affirmatively because the primary aim was to understand who sought treatment for COVID-19 with non–FDA-approved strategies. Participants were asked if they had taken hydroxychloroquine, ivermectin, or one of the antiviral medications approved by the FDA for use in COVID-19. The primary outcome measure was use of hydroxychloroquine or ivermectin; we examined secondarily FDA-approved antiviral use (molnupiravir or nirmatrelvir plus ritonavir) for purposes of comparison.

We collected additional measures for use as covariates. Sociodemographic features were collected by self-report. We collected information on race and ethnicity to facilitate survey weighting to approximate the US adult population; participants selected race and ethnicity from a list that included African American or Black, Asian American, Hispanic, Native American, Pacific Islander, White, or other, with the opportunity to provide a free-text self-description. Because of small cell sizes, Native American, Pacific Islander, and other were collapsed into a single category for analysis. Political affiliation was asked by inquiring, “Generally speaking, do you think of yourself as a…” with Republican, Democrat, Independent, or other as choices.

We assessed COVID-19 vaccine–related misinformation by presenting respondents with statements about the vaccine; they were asked whether they agreed, disagreed, or were not sure about the statement. For analysis, any agreement with any single misinformation item was considered to be endorsing misinformation. Questions are listed in the eAppendix in Supplement 1.

We also measured trust in institutions by asking how much respondents trusted a given group to do the right thing; answers were a lot, some, not too much, or not at all. For primary analysis, we dichotomized this measure to some or a lot vs not too much or not at all, reflecting overall trust or mistrust. This question was asked in relation to physicians and hospitals, the pharmaceutical companies, scientists and researchers, the news media, and social media companies. Trust in Donald Trump, asked in the same way, was also included for purposes of comparison.

We measured conspiracy-mindedness via the 4-item American Conspiracy Thinking Scale,^12^ a briefer and more focused assessment of conspiratorial thought as it relates to the US government than some other more widely used scales.^13^ Each item is scored from strongly disagree (score of 1) to strongly agree (score of 5). A typical question is, “Even though we live in a democracy, a few people will always run things anyway.” Remaining questions are listed in the eAppendix in Supplement 1. This measure yields a total score between 4 (strongly disagree with all conspiratorial statements) and 20 (strongly agree with all conspiratorial statements). Finally, we examined sources of news by asking whether individuals had received any news about COVID-19 in the prior 24 hours from a list of sources (see eAppendix in Supplement 1 for specific question wording).

### Statistical Analysis

Interlocking poststratification weights were generated to better match the US population with respect to 2020 vote choice and turnout, race and ethnicity, age, gender, educational level, region, and living in urban, suburban, or rural areas, using data from the US Census American Community Survey,^14^ applying the survey package in R software, version 4.0 (R Foundation for Statistical Computing).^15^ This approach generates reliable estimates for nonprobability samples.^16^ In light of very small rates of missing data (Table), primary analyses used complete cases; in prior work,^17^ multiple imputation did not meaningfully change results.

**Table.** Characteristics of Individuals Who Did or Did Not Receive Non–Evidence-Based Treatment for COVID-19^a^

Characteristic	No treatment (n = 12 639)	Treatment (n = 799)	Total (N = 13 438)	*P* value
Age, mean (SD), y	42.8 (16.2)	41.1 (14.9)	42.7 (16.1)	.004
Gender				
Female	8706 (68.9)	444 (55.6)	9150 (68.1)	<.001
Male	3933 (31.1)	355 (44.4)	4288 (31.9)	
Educational level				
Graduate degree	1599 (12.7)	100 (12.5)	1699 (12.6)	<.001
College degree	4704 (37.2)	377 (47.2)	5081 (37.8)	
Some college	3284 (26.0)	158 (19.8)	3442 (25.6)	
High school graduate	2645 (20.9)	141 (17.6)	2786 (20.7)	
Some high school or less	407 (3.2)	23 (2.9)	430 (3.2)	
Income, $^b^				
<25 000	2486 (19.7)	114 (14.3)	2600 (19.4)	<.001
25 000-<50 000	3142 (24.9)	156 (19.5)	3298 (24.5)	
50 000-<100 000	4260 (33.7)	312 (39.0)	4572 (34.0)	
≥100 000	2747 (21.7)	217 (27.2)	2964 (22.1)	
Race and ethnicity				
Asian American	918 (7.3)	44 (5.5)	962 (7.2)	<.001
Black	1048 (8.3)	72 (9.0)	1120 (8.3)	
Hispanic	958 (7.6)	110 (13.8)	1068 (7.9)	
Native American	56 (0.4)	3 (0.4)	59 (0.4)	
Pacific Islander	195 (1.5)	14 (1.8)	209 (1.6)	
White	9203 (72.8)	540 (67.6)	9743 (72.5)	
Other^c^	261 (2.1)	16 (2.0)	277 (2.1)	
Urbanicity				
Rural	2612 (20.7)	175 (21.9)	2787 (20.7)	.06
Suburban	7308 (57.8)	429 (53.7)	7737 (57.6)	
Urban	2719 (21.5)	195 (24.4)	2914 (21.7)	
Political affiliation^d^				
Democrat	4311 (34.3)	336 (42.1)	4647 (34.7)	<.001
Independent or other	4701 (37.4)	177 (22.2)	4878 (36.5)	
Republican	3570 (28.4)	286 (35.8)	3856 (28.8)	
Trust in^e^				
Hospitals and physicians	11322 (89.8)	690 (86.9)	12 012 (89.6)	.01
Pharmaceutical industry	6174 (48.9)	496 (62.4)	6670 (49.7)	<.001
Scientists	10 751 (85.2)	617 (77.9)	11 368 (84.8)	<.001
News media	5146 (40.8)	377 (47.3)	5523 (41.2)	<.001
Social media	3235 (25.7)	365 (45.9)	3600 (26.9)	<.001
Donald Trump	4269 (33.9)	456 (57.1)	4725 (35.2)	<.001
Endorsed vaccine misinformation^f^	2201 (21.8)	260 (42.3)	2461 (23.0)	<.001
No. of items endorsed, mean (SD)	0.4 (1.0)	0.9 (1.3)	0.4 (1.0)	<.001
Conspiracy score, mean (SD)^g^	12.7 (3.8)	14.0 (3.7)	12.8 (3.8)	<.001
News source^h^				
Fox News	2152 (21.2)	253 (41.0)	2405 (22.3)	<.001
Facebook	3619 (35.6)	300 (48.6)	3919 (36.3)	<.001
CNN	2320 (22.8)	235 (38.1)	2555 (23.7)	<.001
MSNBC	988 (9.7)	93 (15.1)	1081 (10.0)	<.001
Fox, Facebook, or Newsmax	4897 (48.2)	424 (68.7)	5321 (49.3)	<.001
Prescribing outcomes				
Hydroxychloroquine	0	527 (66.0)	527 (3.9)	NA
Ivermectin	0	440 (55.1)	440 (3.3)	NA
Antiviral medication	793 (6.3)	191 (23.9)	984 (7.3)	<.001

Abbreviation: NA, not applicable.

^a^ Data are presented as number (percentage) of respondents unless otherwise indicated.

^b^ Income not available for 4 individuals who had not received non–evidence-based treatment.

^c^ Includes those who selected the “other” category, which allowed for a free text entry.

^d^ Political affiliation not available for 57 individuals who had not received non–evidence-based treatment.

^e^ Trust not completed for hospitals (n = 34; 29 without treatment and 5 with treatment), pharmaceutical companies (n = 24; 20 without treatment and 4 with treatment), scientists (n = 33; 26 without treatment and 7 with treatment), news media (n = 19; 17 without treatment and 2 with treatment), social media (n = 39; 36 without treatment and 3 with treatment), and Donald Trump (n = 30; 29 with treatment and 1 without treatment).

^f^ Vaccine misinformation not collected for 2720 respondents (2535 with no treatment and 185 with treatment).

^g^ Conspiracy score not collected for 2705 respondents (2520 with no treatment and 185 with treatment).

^h^ News source not collected for 2654 respondents (2472 with no treatment and 182 with treatment).

Primary analysis used logistic regression to examine association of individual characteristics with use of any non–evidence-based medication, alone and then with adjustment for sociodemographic features. For comparison, we also examined association of these features with the use of an FDA-approved antiviral medication. Analyses were conducted with R software, version 4.0 (R Foundation)^15^; because survey weights were applied to approximate national distributions, regression models used svyglm from the survey package (version 4.1-1), with unweighted regression results presented in eTables 2 to 4 in Supplement 2. All tests were 2-tailed and specified an uncorrected 2-sided *P* < .05 as the threshold for statistical significance.

## Results

The full cohort included 13 438 individuals who indicated current or prior COVID-19 illness. Without application of survey weights, the mean (SD) age was 42.7 (16.1) years; 9150 (68.1%) identified as women and 4288 (31.9%) as men. The cohort included 962 (7.2%) who identified as Asian, 1120 (8.3%) as Black, 1068 (7.9%) as Hispanic, 9743 (72.5%) as White, and 545 (4.1%) as other race and ethnicity (including Native American, Pacific Islander, or an “other” category allowing free text entry of some other designation). Additional characteristics of the cohort are summarized in the Table. In all, 799 respondents (5.9%) reported using ivermectin (440 [3.3%], including 305 [69.3%] prescribed by a medical professional) and/or hydroxychloroquine (527 [3.9%], including 455 [86.3%] prescribed by a medical professional). A total of 984 individuals (7.3%) reported use of either molnupiravir (266 [2.0%]) or nirmatrelvir plus ritonavir (821 [6.1%]). Survey-weighted estimates and proportions within each sociodemographic category reporting non–evidence-based medication use are presented in eTable 1 in Supplement 1.

**Figure 1.**
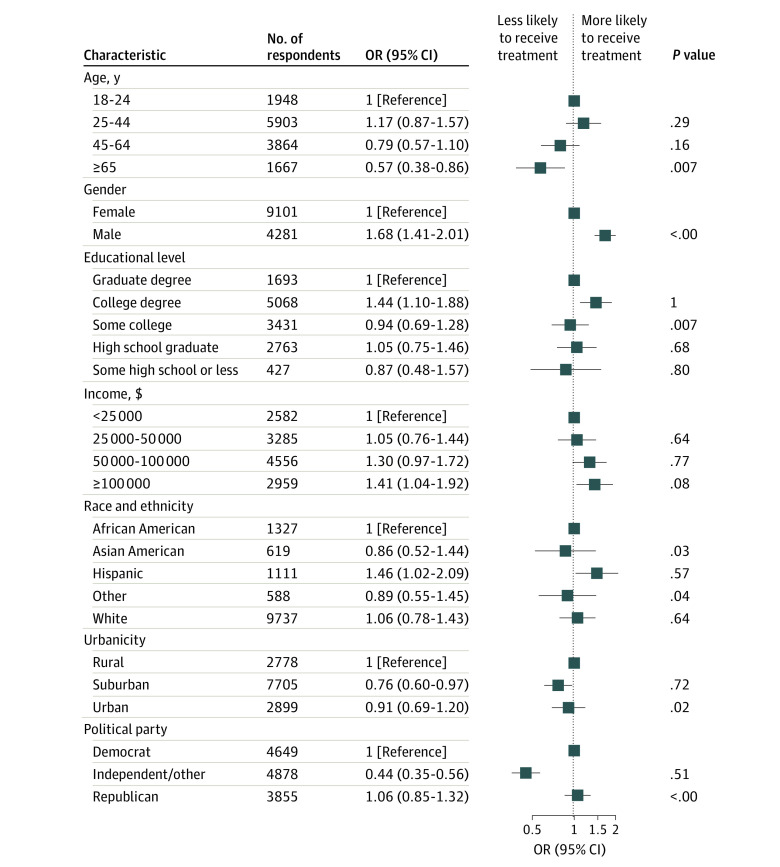
Logistic Regression Model Examining Associations With Receiving Non–Evidence-Based Medication Treatment for COVID-19, Weighted Survey Results Other race and ethnicity includes participants who indicated Native American, Pacific Islander, or other from a list of checkboxes; because of small cell sizes, these categories were collapsed into a single category for analysis. OR indicates odds ratio.

We examined associations between individual sociodemographic characteristics and non–evidence-based medication use. In fully adjusted survey-weighted regression models, being male, having a college degree, having greater income, and being Hispanic were all associated with greater likelihood of using one of these medications; being 65 years or older was associated with lesser likelihood of using one of these (Figure 1). Identifying as politically independent (compared with being a Democrat) was associated with lesser likelihood of use, whereas identifying as being Republican in political orientation was not associated with greater likelihood of use (compared with being a Democrat). By comparison, the pattern was markedly different for FDA-approved antiviral use, with older individuals, those with more education, and those who identified as Democrats compared with independent or Republican being more likely to use evidence-based medication (eFigure 1 in Supplement 1). Regression models without applying survey weights yielded very similar results (eFigure 2 in Supplement 1) for non–evidence-based medication and eFigure 3 in Supplement 1 for FDA-approved antiviral use).

We next considered the extent to which endorsing vaccine-related misinformation was associated with using non–evidence-based medications. In the cohort as a whole, 2461 of 10 718 (23.0%) endorsed at least 1 item of vaccine-related misinformation. This endorsement was associated with a significantly greater likelihood of having used a non–evidence-based treatment in survey-weighted regression models (unadjusted odds ratio [OR], 2.71; 95% CI, 2.22-3.32; adjusted OR, 2.86; 95% CI, 2.28-3.58) (eFigure 4 in Supplement 1). In adjusted but not unadjusted models, misinformation was modestly but significantly associated with receiving prescription antiviral treatment as well (unadjusted OR, 1.00; 95% CI, 0.80-1.24; adjusted OR, 1.36; 95% CI, 1.08-1.71) (eFigure 5 in Supplement 1). Regression models without survey weighting yielded similar results (eFigures 6 and 7 in Supplement 1).

**Figure 2.**
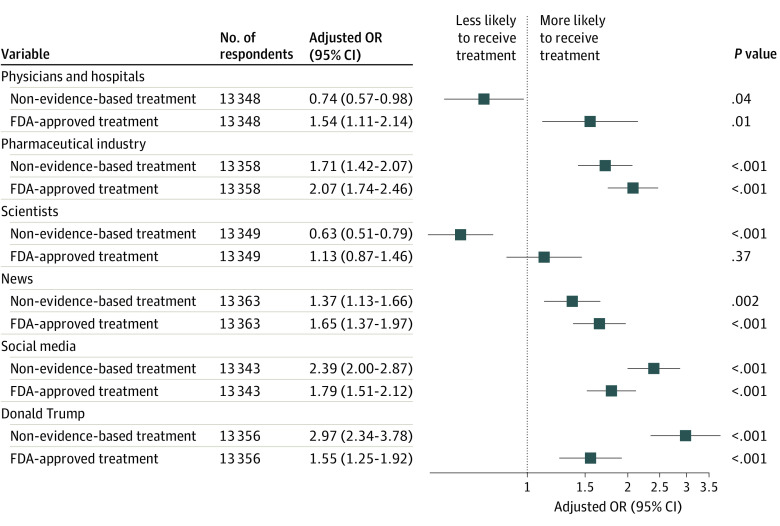
Survey-Weighted, Adjusted Odds Ratios (ORs) of Non–Evidence-Based Treatment Use or US Food and Drug Administration (FDA)–Approved Treatment Use for Respondents Who Described Some or a Lot of Trust in a Given Institution

We also examined trust in institutions, including physicians and hospitals, the pharmaceutical industry, scientists, news media, social media, and Donald Trump, using the same sociodemographic models but adding a term for trust in each institution. Figure 2 illustrates survey-weighted, adjusted ORs of non–evidence-based treatment use for respondents who described some or a lot of trust in a given institution and the corresponding ORs for receiving FDA-approved antiviral treatment, showing differences in patterns of association (for full model results, see eTable 2 in Supplement 2). In particular, trust in hospitals and physicians was associated with diminished likelihood of receiving non–evidence-based treatment (OR, 0.74; 95% CI, 0.56-0.98) but greater likelihood of receiving antiviral treatment (OR, 1.54; 95% CI, 1.11-2.14). A similar pattern was identified wherein an increased trust in scientists corresponded with a diminished likelihood of receiving non–evidence-based treatment (OR, 0.63; 95% CI, 0.51-0.79) and a greater likelihood of receiving antiviral treatment (OR, 1.13; 95% CI, 0.87-1.46). Otherwise, presence of trust was generally associated with greater likelihood of using either non–evidence-based or antiviral treatment, although magnitude of effects varied substantially.

We then considered conspiracy-mindedness for association with use of non–evidence-based medications. In survey-weighted regression models that included the same features as in Figure 1 and added the American Conspiracy Thinking Scale, in addition to trust in scientists, hospitals, and physicians, respondents who reported trust in social media (adjusted OR, 2.39; 95% CI, 2.00-2.87) or Donald Trump (adjusted OR, 2.97; 95% CI, 2.34-3.78), as well as those who scored higher on the American Conspiracy Thinking Scale (unadjusted OR, 1.09; 95% CI, 1.06-1.11; adjusted OR, 1.10; 95% CI, 1.07-1.13), were more likely to have taken non–evidence-based medications. For full model results, see eTable 3 in Supplement 2.

**Figure 3.**
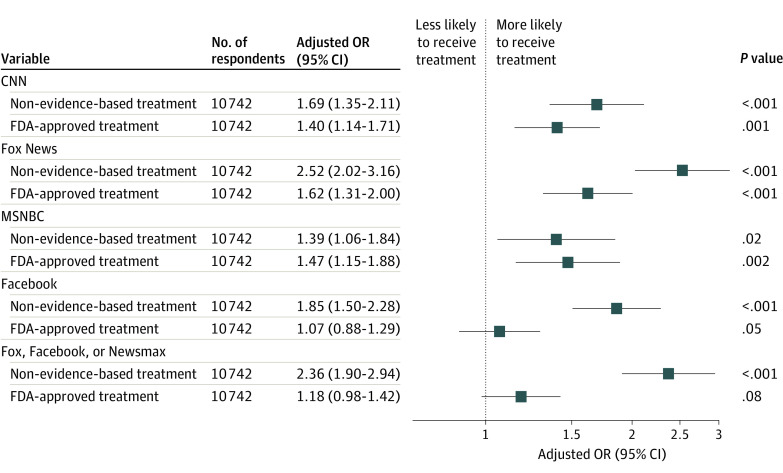
Survey-Weighted, Adjusted Odds Ratios (ORs) of Non–Evidence-Based Treatment Use or US Food and Drug Administration (FDA)–Approved Treatment Use for Respondents Who Reported a Given News Source

Finally, to examine the extent to which information sources about COVID-19 were associated with receiving non–evidence-based medications, we repeated regression models with indicator variables for a subset of cable media outlets (eg, CNN, Fox, and MSNBC) as well as Facebook for consistency with prior work^18^ (Figure 3; eTable 4 in Supplement 2). In general, identifying any of these sources was associated with a significantly greater likelihood of receiving non–evidence-based treatment; similar patterns, albeit of lesser magnitude, were observed for receiving antiviral treatment, with the exception of Facebook as a news source, which was not associated with likelihood of receipt of FDA-approved antiviral treatment.

## Discussion

In this survey study of 13 438 US adults, we found that 799 (5.9%) reported use of at least 1 non–evidence-based prescription for the treatment of COVID-19; by comparison, 984 (7.3%) reported use of an FDA-approved antiviral treatment. Among those using non-evidence-based medications, as much as 30% of individuals using ivermectin and 14% of individuals using hydroxychloroquine reported that they did not receive these interventions from a medical professional, which suggests that studies relying on prescription databases to estimate such use are likely to yield substantial underestimates.

Our results are generally consistent with investigations examining prescribing data for ivermectin^2^ and hydroxychloroquine,^1^ indicating a spike in prescribing early in the pandemic. The large proportion of non–evidence-based treatments not prescribed by a medical professional suggest the limitation of relying on prescription data for quantifying these effects. In prior work,^3^ analyses with county-level data suggested that counties with greater voting for Donald Trump exhibited greater levels of non–evidence-based COVID-19 prescribing. Similarly, other analyses showed that more politically conservative physicians were more likely to favor these interventions.^1,19^ Our study extends these efforts in suggesting that patients’ views are associated with non–evidence-based prescriptions as well. Moreover, it is not solely partisan affiliation that matters. Instead, we found that endorsement of vaccine-related misinformation, mistrust of health care institutions and scientists, and conspiratorial thinking related to politics were all associated with greater likelihood of using non–evidence-based treatments. None of these were simple proxies for political affiliation, which was included as a covariate in all regression models; the effects of trust in institutions persisted after accounting for trust in Donald Trump. This finding counters many narratives that focus on how partisan divides drive gaps in COVID-19–related behaviors.^6^ This distinction is important insofar as altering one’s party identification may be an unrealistic bar, whereas combating misinformation and trying to build institutional trust may be more feasible.^20^

More generally, endorsement of misinformation related to COVID-19 has been shown to decrease the intention to vaccinate against COVID-19, to decrease the belief that it is required for herd immunity, and to correlate with forgoing various COVID-19 prevention behaviors.^7,21^ Such false information is largely spread online and often, though not always, originates as disinformation intentionally spread by political actors and media sources,^22^ as well as illicit actors who profit from touting supposed cures for COVID-19, such as ivermectin.^4^ A substantial minority of the American public endorses false information related to COVID-19, although certain subgroups are more likely to do so, including those who are more religious, who distrust scientists, and who hold stronger political affiliations.^23,24^ There is also a growing body of evidence that cultivating and maintaining trust is a crucial factor in encouraging the public to engage in prosocial health behaviors in the long term in the absence of government mandates.^11,12,13,14^ The extent to which addressing conspiratorial thinking could represent a strategy to address obstacles to public health,^25^ particularly when those messages are conveyed by government entities, merits further investigation.

Our findings regarding the substantial effects of news source on likelihood of using both non–evidence-based medications may also point to potential interventions. In general, cable news sources regardless of perspective were associated with increased odds for both non–evidence-based and FDA-approved antiviral treatment. Facebook did not follow this pattern: odds of non–evidence-based but not FDA-approved treatment were markedly greater with Facebook as a news source. In general, sources of news perceived to be more right-leaning demonstrated a greater gap between non–evidence-based and FDA-approved interventions. Importantly, these models reflected associations after control for political affiliation—that is, the associations we observed are over and above any effects of political party. Our results here are consistent with a prior report suggesting that conservative media outlets were associated with endorsing COVID-19 misinformation,^26^ indicating that these outlets are also associated with nonevidence-based medicine use for COVID-19.

### Limitations

This study has several limitations. Because this was an opt-in internet nonprobability survey, we cannot estimate a response rate because there is no denominator per se; panelists can select surveys from a list of those for which they are eligible. In general, nonprobability sampling as a lower-cost alternative to probability sampling^10^ has been shown to be valid in studies similar to this one^27,28^; we have also demonstrated convergent validity with administrative or other criterion standard data types.^17,18^

Because this was a cross-sectional survey, we cannot infer that misinformation or mistrust causes individuals to pursue non–evidence-based treatments. We also cannot link treatment to a particular time of infection, so it is also possible that some of the apparent associations with treatment actually reflect risk factors for infection itself (ie, factors that might have increased likelihood of illness at a period when non–evidence-based treatments were receiving greater attention). Finally, we relied on self-report for all diagnosis and treatment data. Thus, our results must be seen as complementing studies that draw on health claims or electronic health records in which diagnoses and prescriptions are documented.

## Conclusions

In aggregate, our survey results suggest that the potential harms of misinformation may extend to prescription of ineffective and potentially toxic treatments, rather than simply avoiding health-promoting behaviors, such as vaccination. The extent to which combating misinformation or increasing trust in health care and science may impact choices about treatment merits further investigation, ideally in randomized clinical trials that can establish causation.

## Notes

Supplement 1. **eTable 1.** Survey-Weighted and Unweighted Characteristics of Individuals Who Did, or Did Not, Receive Non–Evidence-Based Treatment for COVID-19 – Row Percentages **eFigure 1.** Logistic Regression Model Examining Associations With Receiving Antiviral Medication Treatment for COVID-19, Weighted Survey Results **eFigure 2.** Logistic Regression Model Examining Associations With Receiving Non-Evidence–Based Medication Treatment for COVID-19, Without Survey Weighting **eFigure 3.** Logistic Regression Model Examining Associations With Receiving FDA-Approved Antiviral Treatment for COVID-19, Without Survey Weighting **eFigure 4.** Logistic Regression Model Including Endorsement of Misinformation Examining Associations With Receiving Non-Evidence–Based Treatment for COVID-19, With Survey Weighting **eFigure 5.** Logistic Regression Model Including Endorsement of Misinformation Examining Associations With Receiving FDA-Approved Antiviral Treatment for COVID-19, With Survey Weighting **eFigure 6.** Logistic Regression Model Including Endorsement of Misinformation Examining Associations With Receiving Non–Evidence-Based Treatment for COVID-19, Without Survey Weighting **eFigure 7.** Logistic Regression Model Including Endorsement of Misinformation Examining Associations With Receiving FDA-Approved Antiviral Treatment for COVID-19, Without Survey Weighting **eAppendix.** Survey Items Incorporated in Analysis

Supplement 2. **eTable 2.** Survey-Weighted Regression Models Examining Association Between Trust in Institutions and Receiving Non–Evidence-Based and FDA-Approved COVID-19 Treatments **eTable 3.** Survey-Weighted Regression Models Examining Association Between ACTS Score and Receiving Non–Evidence-Based and FDA-Approved COVID-19 Treatments **eTable 4.** Survey-Weighted Regression Models Examining Association Between News Source and Receiving Non–Evidence-Based and FDA-Approved COVID-19 Treatments

Supplement 3.
Data Sharing Statement
