# Error in Figure 1

**DOI:** 10.1001/jamahealthforum.2023.4083

**Published:** 2023-10-20

In the Original Investigation titled “Misinformation, Trust, and Use of Ivermectin and Hydroxychloroquine for COVID-19,”^1^ published September 29, 2023, in Figure 1, some of the *P* values were incorrectly aligned with the reported outcomes. This article has been corrected.^1^
