# Supplementary material for: Misinformation, Trust, and Use of Ivermectin and Hydroxychloroquine for COVID-19
Source: JAMA Health Forum. 2023 Sep 29;4(9):e233257. doi: 10.1001/jamahealthforum.2023.3257 (PMC10542734; doi:10.1001/jamahealthforum.2023.3257)
Supplement: Supplement 1. — eTable 1. Survey-Weighted and Unweighted Characteristics of Individuals Who Did, or Did Not, Receive Non–Evidence-Based Treatment for COVID-19 – Row Percentages eFigure 1. Logistic Regression Model Examining Associations With Receiving Antiviral Medication Treatment for COVID-19, Weighted Survey Results eFigure 2. Logistic Regression Model Examining Associations With Receiving Non-Evidence–Based Medication Treatment for COVID-19, Without Survey Weighting eFigure 3. Logistic Regression Model Examining Associations With Receiving FDA-Approved Antiviral Treatment for COVID-19, Without Survey Weighting eFigure 4. Logistic Regression Model Including Endorsement of Misinformation Examining Associations With Receiving Non-Evidence–Based Treatment for COVID-19, With Survey Weighting eFigure 5. Logistic Regression Model Including Endorsement of Misinformation Examining Associations With Receiving FDA-Approved Antiviral Treatment for COVID-19, With Survey Weighting eFigure 6. Logistic Regression Model Including Endorsement of Misinformation Examining Associations With Receiving Non–Evidence-Based Treatment for COVID-19, Without Survey Weighting eFigure 7. Logistic Regression Model Including Endorsement of Misinformation Examining Associations With Receiving FDA-Approved Antiviral Treatment for COVID-19, Without Survey Weighting eAppendix. Survey Items Incorporated in Analysis [file jamahealthforum-e233257-s001.pdf]

Supplementary Online Content

Perlis RH, Trujillo KL, Green J, et al. Misinformation, trust, and use of ivermectin and hydroxychloroquine for COVID-19. *JAMA Health Forum.* Published online September 29, 2023. doi:10.1001/jamahealthforum.2023.3257

eTable. Survey-Weighted and Unweighted Characteristics of Individuals Who Did, or Did Not, Receive Non–Evidence-Based Treatment for COVID-19—Row Percentages
eFigure 1. Logistic Regression Model Examining Associations With Receiving Antiviral Medication Treatment for COVID-19, Weighted Survey Results
eFigure 2. Logistic Regression Model Examining Associations With Receiving Non–Evidence-Based Medication Treatment for COVID-19, Without Survey Weighting
eFigure 3. Logistic Regression Model Examining Associations With Receiving FDA-Approved Antiviral Treatment for COVID-19, Without Survey Weighting
eFigure 4. Logistic Regression Model Including Endorsement of Misinformation Examining Associations With Receiving Non–Evidence-Based Treatment for COVID-19, With Survey Weighting
eFigure 5. Logistic Regression Model Including Endorsement of Misinformation Examining Associations With Receiving FDA-Approved Antiviral Treatment for COVID-19, With Survey Weighting
eFigure 6. Logistic Regression Model Including Endorsement of Misinformation Examining Associations With Receiving Non–Evidence-Based Treatment for COVID-19, Without Survey Weighting
eFigure 7. Logistic Regression Model Including Endorsement of Misinformation Examining Associations With Receiving FDA-Approved Antiviral Treatment for COVID-19, Without Survey Weighting
eAppendix. Survey Items Incorporated in Analysis

This supplementary material has been provided by the authors to give readers additional information about their work.

© 2023 Perlis RH et al. *JAMA Health Forum.*

eTable. Survey-weighted and unweighted characteristics of individuals who did, or did not, receive non–evidence-based treatment for COVID-19—row percentages

	Weighted: No treatment	Treatment	Unweighted: No treatment	Treatment
Age (years) Mean (SD)	44.0 (17.0)	41.0 (15.4)	42.7 (16.2)	41.1 (14.9)
Gender				
Female	95.4	4.6	95.1	4.9
Male	92.3	7.7	91.7	8.3
Education				
Graduate Degree	93.9	6.1	94.1	5.9
College Degree	91.4	8.6	92.6	7.4
Some College	95.0	5.0	95.4	4.6
High School Graduate	94.7	5.3	94.9	5.1
Some High School or Less	95.6	4.4	94.6	5.4
Income^				
< $25,000	95.5	4.5	95.6	4.4
$25,000 – less than $50,000	95.0	5.0	95.3	4.7
$50,000 – less than $100,000	93.3	6.7	93.2	6.8
$100,000 or more	92.3	7.7	92.7	7.3
Race and Ethnicity				
African American	94.4	5.6	94.2	5.8
Asian American	94.7	5.3	95.0	5.0
Hispanic	91.9	8.1	90.1	9.9
Native American	97.4	2.6	96.9	3.1
Other	95.1	4.9	94.3	5.7
Pacific Islander	93.9	6.1	92.9	7.1
White	94.3	5.7	94.4	5.6
Urbanicity				
Rural	93.7	6.3	93.7	6.3
Suburban	94.6	5.4	94.5	5.5
Urban	92.9	7.1	93.3	6.7
Political Affiliation #				
Democrat	92.4	7.6	92.8	7.2
Independent/Other	96.5	3.5	96.4	3.6
Republican	92.6	7.4	92.6	7.4
Trust in…				
Hospitals and doctors	92.5	7.5	94.3	5.7
Pharmaceutical industry	94.3	5.7	92.6	7.4
Scientists	92.9	7.1	94.6	5.4
News media	89.7	10.3	93.2	6.8
Social media	90.5	9.5	89.9	10.1
Donald Trump	89.2	10.8	90.3	9.7
Endorsed vaccine misinformation (a)	89.4	10.6	89.4	10.6

© 2023 Perlis RH et al. *JAMA Health Forum.*

	Weighted: No treatment	Treatment	Unweighted: No treatment	Treatment
Number of items endorsed Mean (SD)	0.4 (1.0)	0.9 (1.3)	0.4 (1.0)	0.9 (1.3)
Conspiracy Score Mean (SD) (b)	12.8 (3.9)	14.0 (3.7)	12.7 (3.8)	14.0 (3.7)
News Source (c)				
Fox News	90.0	10.0	89.5	10.5
Facebook	91.6	8.4	92.3	7.7
CNN	91.1	8.9	90.8	9.2
MSNBC	91.9	8.1	91.4	8.6
Fox, Facebook, or Newsmax	91.8	8.2	92.0	8.0
**Prescribing outcomes**				
Hydroxychloroquine	0.0	100.0	0.0	100.0
Ivermectin	0.0	100.0	0.0	100.0
Antiviral medication	80.3	19.7	80.6	19.4

^ Income not available for 4 individuals who had not received non–evidence-based treatment
# Political affiliation not available for 57 individuals who had not received non–evidence-based treatment
* Trust not completed for hospitals (n=34; 29 without treatment/5 with); pharma (24; 20/4); scientists (33; 26/7); news media (19; 17/2) social media (39; 36/3) and Donald Trump (30; 29/1).
a. Vaccine misinformation not collected for n=2720 (2535 with no treatment, 185 with treatment)
b. Conspiracy score not collected for n=2705, including 2520 with no treatment and 185 with treatment.
c. News source not collected for n=2654, including 2472 with no treatment and 182 with treatment

© 2023 Perlis RH et al. *JAMA Health Forum.*

eFigure 1. Logistic regression model examining associations with receiving antiviral medication treatment for COVID-19, weighted survey results

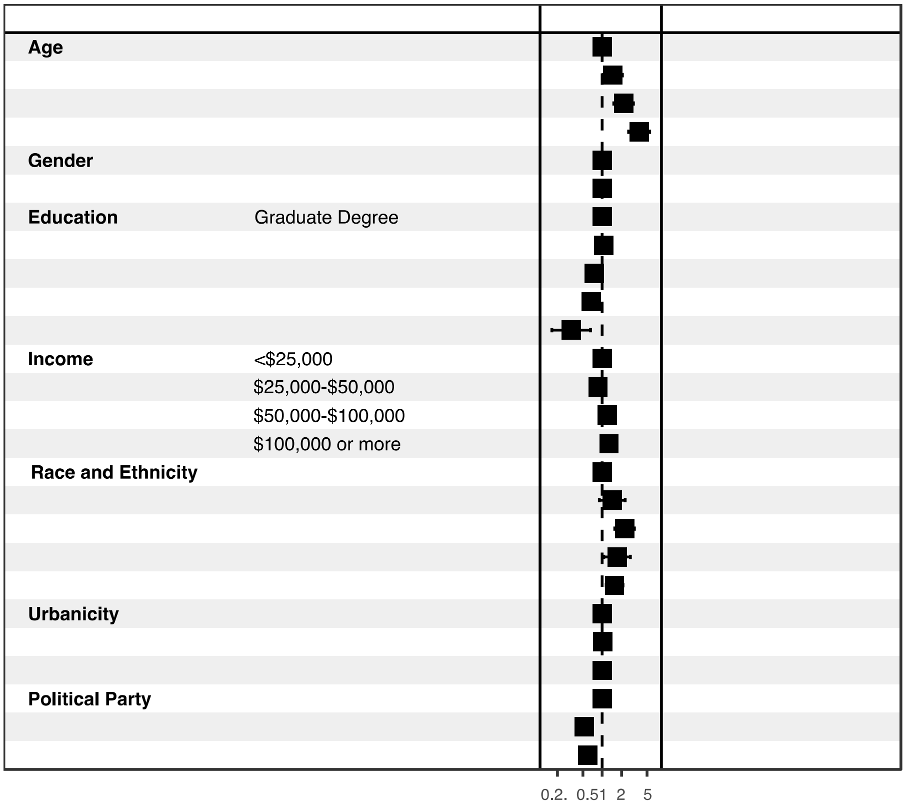

© 2023 Perlis RH et al. *JAMA Health Forum.*

eFigure 2. Logistic regression model examining associations with receiving non–evidence-based medication treatment for COVID-19, without survey weighting

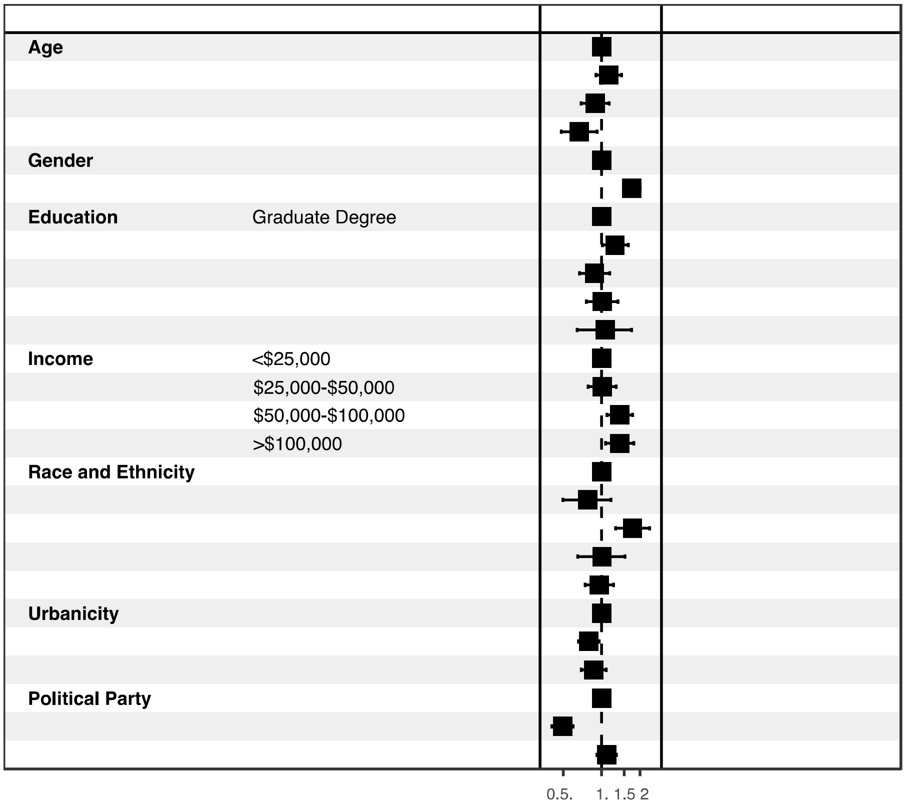

Other race and ethnicity includes Native American, Pacific Islander, or ‘Other’ checkbox selected during survey completion

© 2023 Perlis RH et al. *JAMA Health Forum.*

eFigure 3. Logistic regression model examining associations with receiving FDA-approved antiviral treatment for COVID-19, without survey weighting

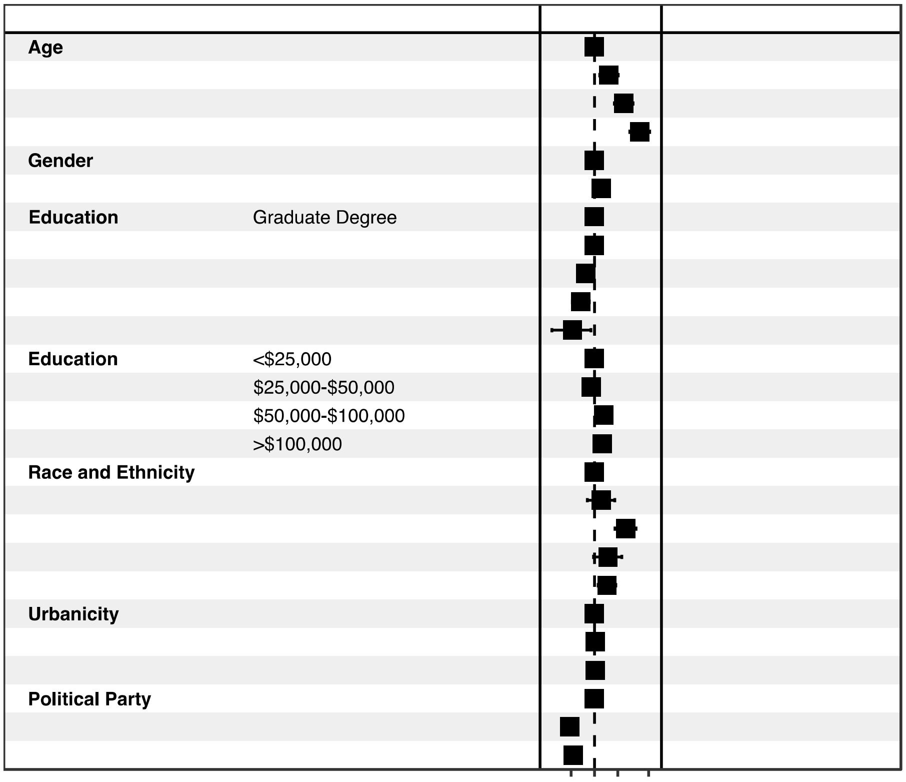

Other race and ethnicity includes Native American, Pacific Islander, or ‘Other’ checkbox selected during survey completion

© 2023 Perlis RH et al. *JAMA Health Forum.*

eFigure 4. Logistic regression model including endorsement of misinformation examining associations with receiving non–evidence-based treatment for COVID-19, with survey weighting

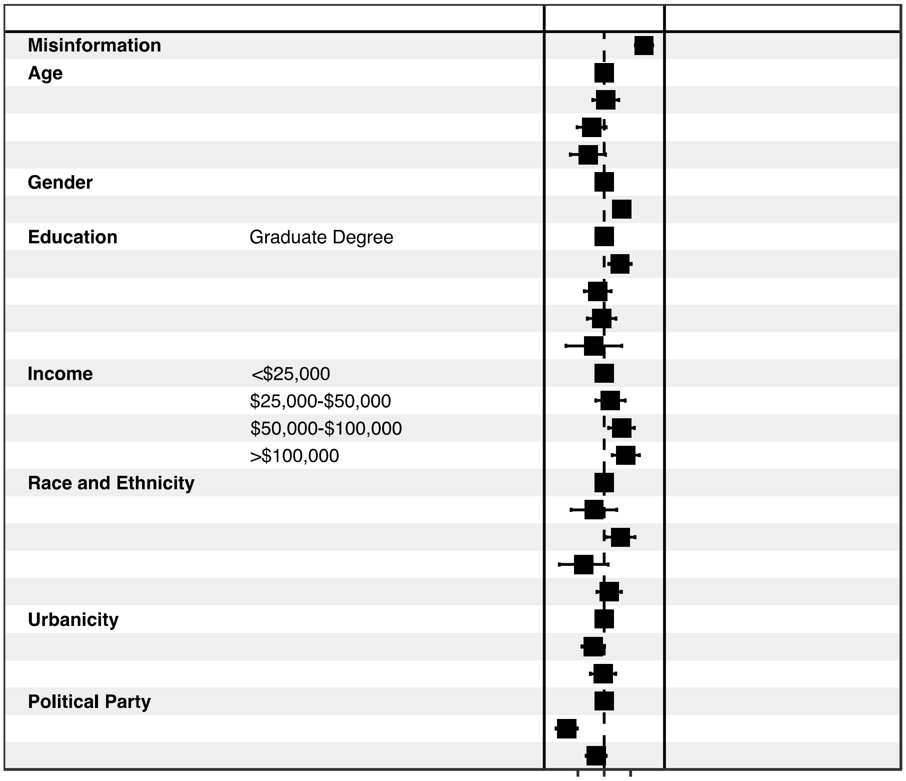

Other race and ethnicity includes Native American, Pacific Islander, or ‘Other’ checkbox selected during survey completion

© 2023 Perlis RH et al. *JAMA Health Forum.*

eFigure 5. Logistic regression model including endorsement of misinformation examining associations with receiving FDA-approved antiviral treatment for COVID-19, with survey weighting

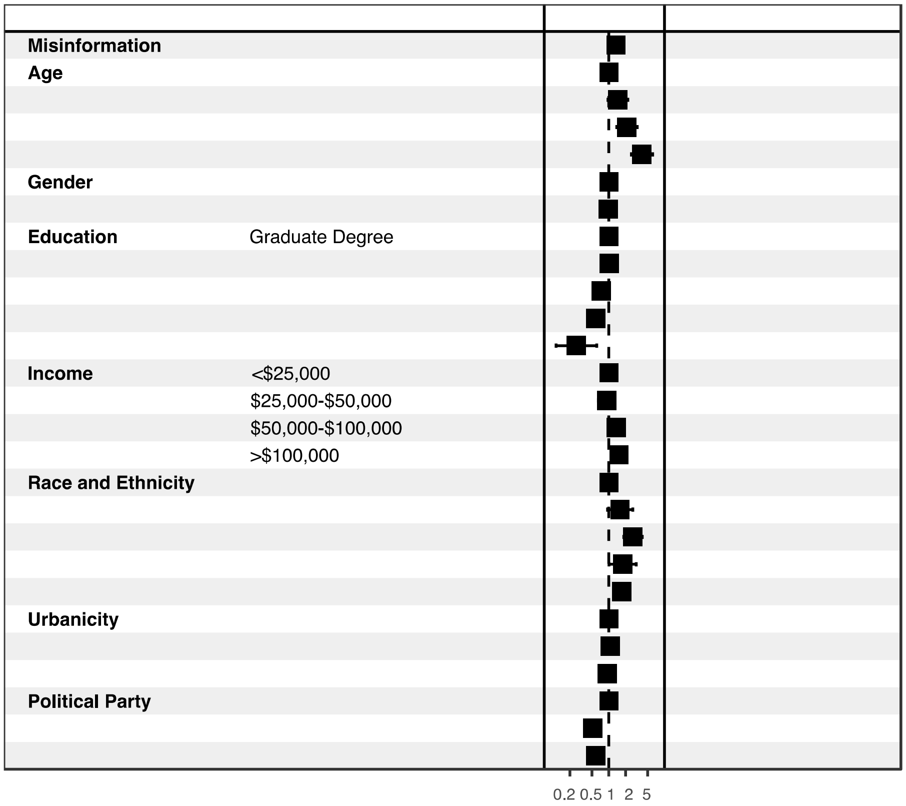

Other race and ethnicity includes Native American, Pacific Islander, or ‘Other’ checkbox selected during survey completion

© 2023 Perlis RH et al. *JAMA Health Forum.*

eFigure 6. Logistic regression model including endorsement of misinformation examining associations with receiving non–evidence-based treatment for COVID-19, without survey weighting

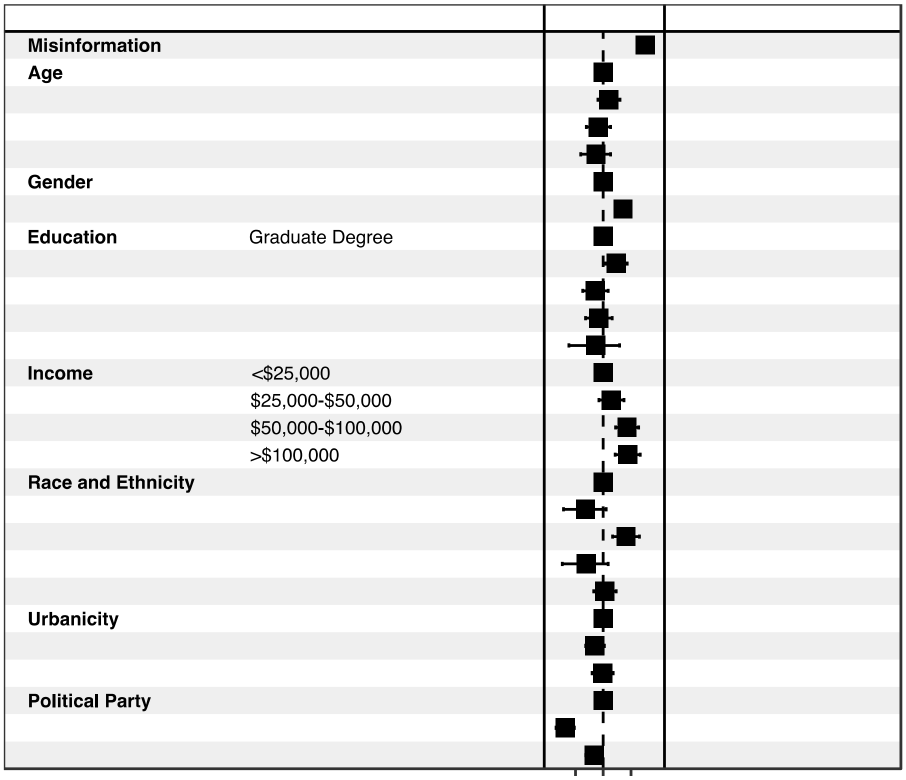

Other race and ethnicity includes Native American, Pacific Islander, or ‘Other’ checkbox selected during survey completion

© 2023 Perlis RH et al. *JAMA Health Forum.*

eFigure 7. Logistic regression model including endorsement of misinformation examining associations with receiving FDA-approved antiviral treatment for COVID-19, without survey weighting

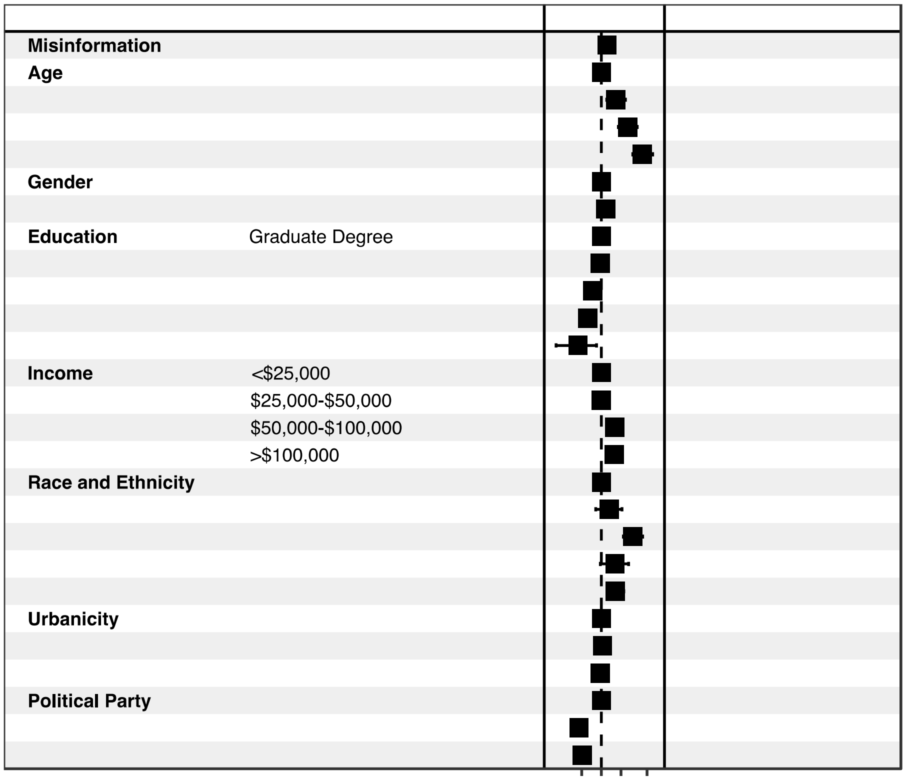

Other race and ethnicity includes Native American, Pacific Islander, or ‘Other’ checkbox selected during survey completion

© 2023 Perlis RH et al. *JAMA Health Forum.*

eAppendix. Survey items incorporated in analysis

Covid diagnosis:

[covid] Have you ever been diagnosed with coronavirus (COVID-19)?
1 = Yes, I was diagnosed by a medical professional
2 = No, I was not diagnosed but I think I may have it now
3 = No, I was not diagnosed but I think I had it previously and recovered
4 = No, I was not diagnosed and I do not think I ever had it
5 = I am not sure

Covid treatment:

[cov_meds] Have you ever taken any of the following medications in order to treat or prevent COVID-19? (Please select all that apply)
[cov_meds_2] Hydroxychloroquine (HCQ)
[cov_meds_3] Ivermectin
[cov_meds_4] Molnupiravir
[cov_meds_5] Paxlovid
[cov_meds_6] None of the above

1 = Selected

[cov_meds_doc] Were the following medications prescribed to you by a medical professional? -
[cov_meds_doc_2] Hydroxychloroquine (HCQ)
[cov_meds_doc_3] Ivermectin
[cov_meds_doc_4] Molnupiravir
[cov_meds_doc_5] Paxlovid

1 = Yes
2 = No

Misinformation about COVID-19 vaccine:

[vac_fn] Below are some statements about the COVID-19 vaccines that are currently being distributed. To the best of your knowledge, are those statements accurate or inaccurate? - The COVID-19 vaccines
[vac_fn_1] will alter people's DNA.
[vac_fn_2] contain microchips that could track people.
[vac_fn_3] contain the lung tissue of aborted fetuses.
[vac_fn_4] can cause infertility, making it more difficult to get pregnant.
[vac_fn_10] contain a bioluminescent marker used to trace people

© 2023 Perlis RH et al. *JAMA Health Forum.*

1 = Accurate
2 = Inaccurate
3 = Not sure

Trust in institutions:

[pol_trust1] How much do you trust the following people and organizations to do what is right?
[pol_trust1_5] Donald Trump

[pol_trust2_1] Hospitals and doctors
[pol_trust2_2] Pharmaceutical companies
[pol_trust2_3] Scientists and researchers
[pol_trust2_6] The news media
[pol_trust2_7] Social media companies

4 = A lot
3 = Some
2 = Not too much
1 = Not at all

American Conspiratorial Thinking Scale (ACTS):

[conspiracy] How much do you agree or disagree with the following statements? -
[conspiracy_1] Even though we live in a democracy, a few people will always run things anyway.
[conspiracy_2] The people who really 'run' the country are not known to the voters
[conspiracy_3] Big events like wars, the current recession, and the outcomes of elections are controlled by small groups of people who are working in secret against the rest of us.
[conspiracy_4] Much of our lives are being controlled by plots hatched in secret places

5 = Strongly agree
4 = Somewhat agree
3 = Neither agree nor disagree
2 = Somewhat disagree
1 = Strongly disagree

Sources of information about current affairs:

[pol_news2] In the last 24 hours, did you get any news or information about politics and current affairs from the following sources? (Please select all that apply)
[pol_news2_1] Facebook (website or app)

© 2023 Perlis RH et al. *JAMA Health Forum.*

[pol_news4] In the last 24 hours, did you get any news or information about politics and current affairs from the following sources, either online or offline? (Please select all that apply)
[pol_news4_3] CNN
[pol_news4_6] Fox News
[pol_news4_9] MSNBC
[pol_news4_13] Newsmax

© 2023 Perlis RH et al. *JAMA Health Forum.*
