# Supplementary material for: Misinformation, Trust, and Use of Ivermectin and Hydroxychloroquine for COVID-19
Source: JAMA Health Forum. 2023 Sep 29;4(9):e233257. doi: 10.1001/jamahealthforum.2023.3257 (PMC10542734; doi:10.1001/jamahealthforum.2023.3257)
Supplement: Supplement 3. — Data Sharing Statement [file jamahealthforum-e233257-s003.pdf]

Data Sharing Statement

Perlis. Misinformation, Trust, and Use of Ivermectin and Hydroxychloroquine for COVID-19. *JAMA Health Forum*. Published September 29, 2023. doi:10.1001/jamahealthforum.2023.3257

Data
Data available: No

Additional Information
Explanation for why data not available: Deidentified data for the full survey will be shared in 2023 but release date not yet set.
